# Urinary [TIMP-2]•[IGFBP7], TIMP-2, IGFBP7, NGAL, and L-FABP for the prediction of acute kidney injury following cardiovascular surgery in Japanese patients

**DOI:** 10.1007/s10157-025-02671-2

**Published:** 2025-04-07

**Authors:** Hideki Iwata, Taro Horino, Yuki Osakabe, Satoshi Inotani, Keisuke Yoshida, Keita Mitani, Yutaka Hatakeyama, Yujiro Miura, Yoshio Terada, Takashi Kawano

**Affiliations:** 1 Department of Anesthesiology and Intensive Care Medicine, Kochi Medical School, Kochi University, Kohasu, Oko-cho, Nankoku, Kochi 783-8505 Japan; 2 Department of Endocrinology, Metabolism and Nephrology, Kochi Medical School, Kochi University, Kohasu, Oko-cho, Nankoku, Kochi 783-8505 Japan; 3 Department of Cardiovascular Surgery, Kochi Medical School, Kochi University, Kohasu, Oko-cho, Nankoku, Kochi 783-8505 Japan; 4 Centre of Medical Information Science, Kochi Medical School, Kochi University, Kohasu, Oko-cho, Nankoku, Kochi 783-8505 Japan

**Keywords:** Acute kidney injury, Biomarker, Cardiovascular surgery, TIMP-2, IGFBP7, NGAL, L-FABP

## Abstract

**Background:**

Acute kidney injury (AKI) following cardiac surgery is common and is associated with poor outcomes. The combination of urinary tissue inhibitor of metalloproteinase 2 (TIMP-2) and insulin-like growth factor-binding protein 7 (IGFBP7) is a strong predictor of AKI after cardiac surgery. However, most studies have focused on non-Asian populations, and comparisons with other AKI biomarkers or the optimal timing for measurement have yet to be explored.

**Methods:**

We prospectively enrolled adult patients at Kochi Medical School Hospital in Kochi, Japan, to assess the predictive values of [TIMP-2]•[IGFBP7], TIMP-2, IGFBP7, neutrophil gelatinase-associated lipocalin (NGAL), and liver fatty acid-binding protein (L-FABP) measured preoperatively and at 2, 4, 6, and 8 h, as well as on day 1 and day 2 after postoperative intensive care unit (ICU) admission, using receiver operating characteristic curve (ROC) analysis.

**Results:**

Of the 38 patients, 13 (34.2%) developed AKI: seven (18.4%) with stage 1, four (10.5%) with stage 2, and two (5.2%) with stage 3. ROC analysis showed that the area under the curve (AUC) for predicting any stage of AKI peaked at 0–4 h, with the highest value at 2 h after ICU admission. Among the biomarkers, [TIMP-2]•[IGFBP7] showed the best AUC at 2 h after ICU admission, followed by TIMP-2, IGFBP7, L-FABP, and NGAL.

**Conclusions:**

Our study demonstrated the good predictive performance of urine biomarkers, including [TIMP-2]•[IGFBP7], TIMP-2, IGFBP7, NGAL, and L-FABP, for any stage of cardiac surgery-associated AKI (CSA–AKI). The combination of TIMP-2 and IGFBP7 measured 2 h after postoperative ICU admission effectively predicted CSA–AKI, identifying patients at higher risk.

**Supplementary Information:**

The online version contains supplementary material available at 10.1007/s10157-025-02671-2.

## Introduction

Acute kidney injury (AKI) is a significant global health issue, associated with elevated morbidity and mortality [1, 2]. AKI is a critical complication in patients within specific hospital settings, such as intensive care units (ICUs), cardiac surgery, oncology, and transplant centres, where its prevalence can sometimes exceed 50% [1, 3]. The incidence of cardiac surgery-associated AKI (CSA–AKI) has been reported to range from 5 to 42% [4, 5], with AKI occurring in up to one-third of patients following cardiac surgery [6]. Recent meta-analyses report a pooled overall incidence rate of 22.3%, with 13.6%, 3.8%, and 2.7% for AKI stages 1, 2, and 3, respectively [7]. CSA–AKI is independently associated with increased morbidity and mortality [4].

The global standard for diagnosing AKI was proposed by Kidney Disease: Improving Global Outcomes (KDIGO) in 2012. It is based on an increase in serum creatinine (SCr) levels and a decrease in urine output (UO) over time [8]. However, therapeutic intervention is often initiated late, as changes in SCr levels typically result from renal tissue damage [9, 10]. The identification of novel AKI biomarkers holds significant potential to enhance the diagnostic approach and treatment of AKI [1]. Kidney "damage" markers could provide valuable "lead time" to address AKI, and "stress" markers may be even more effective [1, 9].

The first-generation AKI biomarkers, such as damage markers like neutrophil gelatinase-associated lipocalin (NGAL) and L-type fatty acid-binding proteins (L-FABP), have been developed over the past 20 years, but they have limitations in specificity and sensitivity, particularly in patients with comorbid conditions [1]. In contrast, second-generation AKI biomarkers, including stress markers such as tissue inhibitor of metalloproteinase 2 (TIMP-2) and insulin-like growth factor-binding protein 7 (IGFBP7), have been developed primarily in the past 10 years [11]. Both TIMP-2 and IGFBP7 have been implicated in G1 cell cycle arrest [1]. For short durations, cell cycle arrest is likely protective, preventing cells from entering the cell cycle during periods of imminent or ongoing injury [12]. Early activation of these cell cycle arrest markers serves as an alarm signal, indicating that something is wrong.

The Sapphire Study demonstrated that TIMP-2 and IGFBP7 outperform previously identified biomarkers, such as NGAL and L-FABP [11]. Furthermore, [TIMP-2]•[IGFBP7], the multiplication index of these two biomarkers, was more effective in diagnosing AKI than either TIMP-2 or IGFBP7 alone and was significantly superior to all previously identified AKI biomarkers [11]. This test is available in many countries, particularly in developed Western nations, but not in some Asian countries, including Japan. To the best of our knowledge, studies on biomarkers in Asian populations are limited. Moreover, the superiority of one biomarker over another, and the potential advantages of biomarkers over clinical models, remain uncertain.

The original concept of AKI encompasses not only moderate-to-severe AKI (stages 2–3) but also mild AKI (stage 1), which significantly alters the prognosis of patients [8]. Although CSA–AKI, like AKI in other settings, has a higher incidence of stage 1 than stages 2 and 3, most previous studies on AKI biomarkers in CSA–AKI have focused solely on stages 2 and 3, which are classified as moderate to severe AKI [11, 13, 14]. As a result, the utility of AKI biomarkers for diagnosing AKI overall (stages 1 to 3) has not been sufficiently validated. Furthermore, the primary endpoints of previous studies on CSA–AKI have varied widely, ranging from 12 h to 7 days, and the measurement time points for AKI biomarkers have also varied, ranging from 0 to 24 h [15]. To date, no comprehensive studies have been conducted to identify the most appropriate AKI biomarkers for diagnosis or the optimal timing for their measurement.

Given the limited number of studies on AKI biomarkers in Asian populations, this study aimed to evaluate the predictive utility of [TIMP-2]•[IGFBP7], TIMP-2, IGFBP7, NGAL, and L-FABP in a Japanese cohort undergoing cardiovascular surgery. Additionally, we sought to determine the optimal timing for measuring urinary biomarkers to diagnose CSA–AKI.

## Materials and methods

### Study design and participants

This prospective observational study, approved by the Institutional Review Board (IRB No.: 28–103), enrolled adult patients undergoing cardiovascular surgery at Kochi Medical School Hospital between July 2022 and October 2023. All enrolled patients provided written informed consent. The exclusion criteria were age less than 18 years, chronic dialysis, and prior renal transplantation.

### Demographic and clinical information

The clinical data for the study were extracted retrospectively from hospital records by trained doctors and research nurses. These data included patient demographics, detailed medical history, surgical procedures, and laboratory test results, such as SCr and UO for AKI assessment. The estimated glomerular filtration rate (eGFR) was calculated using the Japanese equation for eGFR based on the SCr data [16]. Additionally, standardised definitions were applied for the collection of clinical information: for diabetes, we used the American Diabetes Association criteria [17], and laboratory tests were assessed using standardised enzymatic methods with quality control protocols.

### Measurement of urinary biomarkers: Urinary TIMP-2, IGFBP7, [TIMP-2]•[IGFBP7], NGAL, and L-FABP

Urine samples were collected preoperatively, at 2-h intervals from 0 to 8 h post-ICU admission, and at 1 and 2 days post-ICU admission for biomarker analysis. The urine samples were centrifuged after being stored at 4 °C for up to 12 h following collection. [TIMP-2]•[IGFBP7] was measured in urine supernatants using the VITROS NephroCheck immunoassay on a VITROS XT7600 immunodiagnostic system (Ortho Clinical Diagnostics) according to the manufacturer’s instructions. NGAL levels in the urine supernatants were measured using the ARCHITECT urine NGAL assay (Abbott), a chemiluminescent microparticle immunoassay, following the manufacturer’s instructions. L-FABP was measured in urine supernatants using the LUMIPULSE urine L-FABP assay (Fujirebio), a chemiluminescent enzyme immunoassay, according to the manufacturer’s instructions. Certified laboratory technicians, blinded to the clinical data, performed the analyses.

### Endpoints

The primary endpoint was the occurrence of AKI, defined by KDIGO criteria [8], which include an increase in SCr of ≥ 0.3 mg/dL within 48 h, a 1.5-fold increase within 7 days, or UO of < 0.5 mL/kg/h for ≥ 6 h. SCr levels in blood samples collected at hospital admission were used as baseline values for AKI staging.

### Statistical analysis

All analyses were performed using R software (version 4.1.2; R Foundation for Statistical Computing, Vienna, Austria). Patient demographics are presented as means ± standard deviation (SD) for continuous variables and as counts with percentages for categorical variables. Continuous variables were compared using the Mann–Whitney U test, while categorical variables were analysed using Fisher’s exact test. Statistical significance was set at p < 0.05.

The analysis of [TIMP-2]•[IGFBP7], TIMP-2, IGFBP7, L-FABP, and NGAL time-course data was conducted independently at each time point using Mann–Whitney U tests. The differences between each time point and the preoperative baseline within each group, as well as the differences between groups at each time point, were tested.

Sensitivity and specificity analyses were performed using receiver operating characteristic (ROC) curves and R software. The predictive value of these variables for severe AKI was evaluated using ROC analysis and the corresponding area under the curve (AUC). Based on the incidence of CSA–AKI reported in many previous studies and reviews, the predicted incidence in this study was 30%. The sample size required for ROC analysis was calculated based on an AUC predictive value of 0.8, a power of 0.9, a prevalence of 30%, and a significance level of 0.05, suggesting the need for 10 patients with AKI, 23 patients without AKI, and a total study population of 33. The results of this study met all of these calculation parameters. The diagnostic value of a biomarker was defined as excellent with an AUC > 0.9, good with an AUC of 0.75–0.9, poor with an AUC of 0.50–0.75, and without any diagnostic value with an AUC < 0.5 [18].

## Results

### Patient demographics and baseline characteristics

**Fig. 1:**
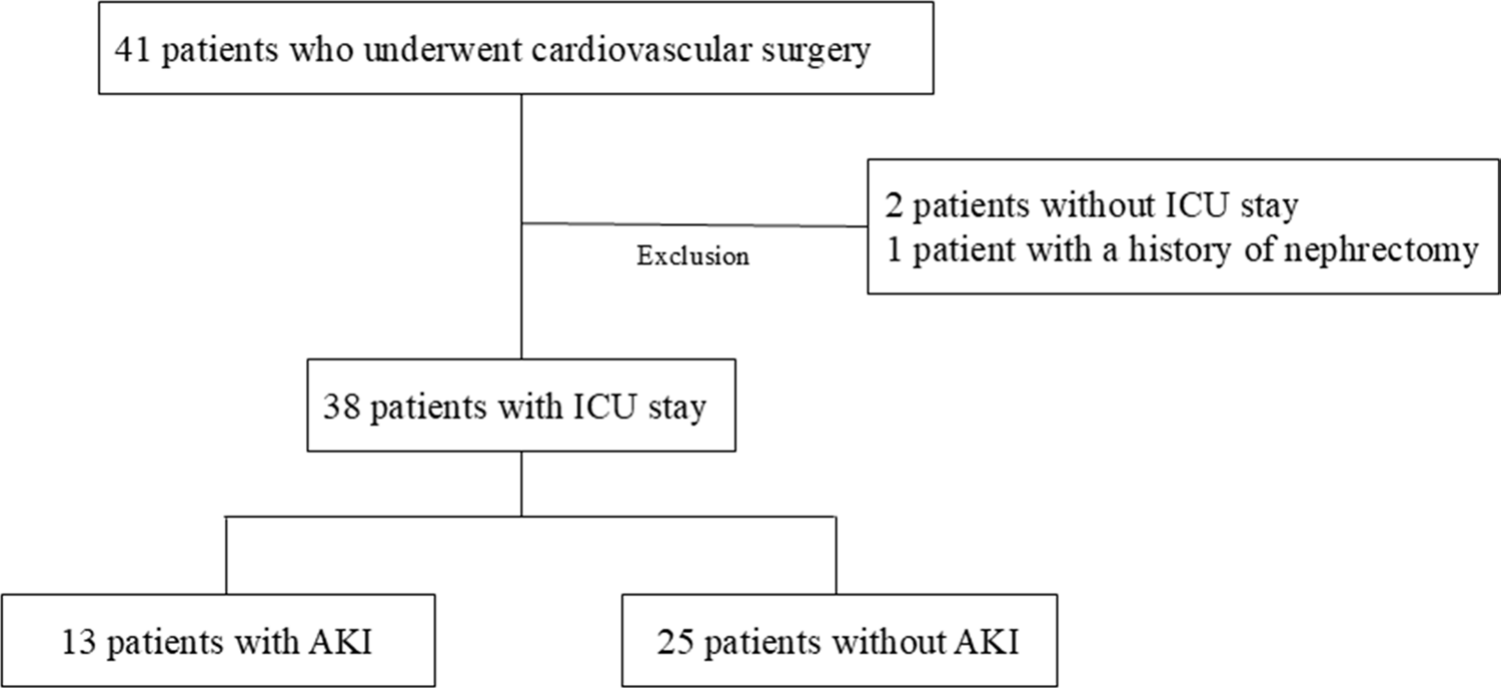
Flowchart illustrating the selection process for the study cohort, outlining the inclusion and exclusion criteria and the number of participants at each stage

A flowchart illustrating the cohort selection process used in this study is presented in Fig. 1. We enrolled 41 patients who had undergone cardiovascular surgery. Two patients were not admitted to the ICU after surgery, and one patient had previously undergone nephrectomy and had only one kidney. After excluding these three patients, 38 were included in the final cohort. Thirteen patients (34.2%) developed AKI: seven (18.4%) had stage 1, four (10.5%) had stage 2, and two (5.2%) had stage 3 AKI. Among the 13 AKI cases, 8 patients met the SCr criteria, all within the first postoperative day. UO criteria identified 10 patients, with 8 diagnosed by the 6th postoperative hour and 2 by the 7th hour. Five patients fulfilled both the SCr and UO criteria.

Table 1 summarises the descriptive statistics for the entire patient group and the subgroups with and without AKI, along with the* p*-values for subgroup differences. In the entire patient group (n = 38), the mean age was 73.9 ± 9.6 years, and 36.8% were women. The body mass index was 24.3 ± 4.5 kg/m^2^. The prevalence of pre-existing hypertension, diabetes mellitus, and heart failure was 89.5%, 34.2%, and 18.4%, respectively. Baseline SCr and eGFR were 1.0 ± 0.3 mg/dL and 54.3 ± 17.8 mL/min/1.73 m^2^, respectively. The urine protein excretion level was 432.9 ± 1,236.7 mg/gCr. No significant differences in age, sex, or body mass index were observed between patients with and without AKI. Patients with AKI had a higher prevalence of pre-existing heart failure (38.5% vs. 8%, *p* = 0.034) and worse baseline SCr and estimated glomerular filtration rate (eGFR) (1.2 ± 0.3 mg/dL vs 0.9 ± 0.3 mg/dL, *p* = 0.019; 44.9 ± 14.8 mL/min/1.73 m^2^ vs 59.1 ± 17.2 mL/min/1.73 m^2^, *p* = 0.021). No differences were observed in urine protein excretion levels between the subgroups.

**Table 1:** Characteristics of the entire patient group and subgroups with and without AKI

	All patients (n = 38)	AKI (n = 13)	no AKI (n = 25)	*p*-value
Preoperative (baseline)	73.9 (9.6)	74.0 (8.9)	73.9 (10.0)	0.988
Age (years old), mean (SD)	73.9 (9.6)	74.0 (8.9)	73.9 (10.0)	0.988
Sex: women, n (%)	14 (36.8%)	5 (38.5%)	9 (36.0%)	1.000
Body mass index (kg/m^2^), mean (SD)	24.3 (4.5)	23.4 (2.9)	24.8 (5.0)	0.329
ASA PS, mean (SD)	2.4 (0.5)	2.5 (0.5)	2.4 (0.5)	0.305
Hypertension, n (%)	34 (89.5%)	10 (76.9%)	24 (96.0%)	0.107
Diabetes mellitus, n (%)	13 (34.2%)	7 (53.9%)	6 (24.0%)	0.084
Heart failure, n (%)	7 (18.4%)	5 (38.5%)	2 (8.0%)	0.034
eGFR (mL/min/1.73m^2^), mean (SD)	54.3 (17.8)	44.9 (14.8)	59.1 (17.2)	0.021
SCr (mg/dL), mean (SD)	1.0 (0.3)	1.2 (0.3)	0.9 (0.3)	0.019
Urine protein excretion (mg/gCr), mean (SD)	432.9 (1,236.7)	553.4 (1,289.7)	367.6 (1,201.9)	0.987
Perioperative				
Cardiopulmonary bypass, *n* (%)	15 (39.5%)	8 (61.5%)	7 (28.0%)	0.079
Anaesthesia time (min), mean (SD)	436.5 (208.8)	541.0 (209.1)	382.2 (186.8)	0.015
Surgery time (min), mean (SD)	359.7 (200.4)	449.4 (205.8)	313.0 (180.7)	0.023
Bleeding volume (mL), mean (SD)	1,596.0 (1,602.2)	2,199.6 (1,271.7)	1,282.2 (1,665.3)	0.015
Intake and output (mL), mean (SD)	2,171.9 (1658.8)	2,658.2 (2,098.9)	1,919.1 (1305.7)	0.406
Clinical outcomes				
Hospital LOS (days), mean (SD)	26.7 (31.6)	31.6 (29.1)	24.1 (32.5)	0.064
ICU LOS (days), mean (SD)	4.9 (5.9)	8.0 (8.7)	3.3 (2.3)	0.003
Died during hospitalisation, n (%)	2 (5.3%)	2 (15.4%)	0 (0.0%)	0.111
Incidental haemodialysis induction, n (%)	0 (0.0%)	0 (0.0%)	0 (0.0%)	–

*AKI* acute kidney injury, *ASA PS* American Society of Anaesthesiologists Physical Status, *eGFR* estimated glomerular filtration rate, *SCr* serum creatinine, *LOS* length of stay, *ICU* Intensive Care Unit

ROC analysis revealed that AUCs of SCr, and urine protein excretion were 0.737, and 0.497, respectively, which are poor predictive values (AUC 0.5–0.75) for AKI (Supplementary Fig. 1).

### Perioperative data

Cardiopulmonary bypass was used in 39.5% of patients, with no difference between subgroups. Cardiopulmonary bypass surgeries included two aortic valve surgeries, five mitral valve surgeries, four aortic surgeries, three coronary artery bypass grafting procedures, and one pericardial fenestration. Surgeries without cardiopulmonary bypass included one off-pump coronary artery bypass graft, seven open aortic repairs, fourteen endovascular aortic repairs, and one axillofemoral bypass. Patients with AKI had significantly longer anaesthesia and surgery times than those without AKI (541.0 ± 209.1 min vs. 382.2 ± 186.8 min, *p* = 0.015; 449.4 ± 205.8 min vs. 313.0 ± 180.7 min, *p* = 0.023). Bleeding volume was higher in patients with AKI (2,199.6 ± 1,271.7 mL vs. 1,282.2 ± 1,665.3 mL, *p* = 0.015). The fluid intake and output balance was 2,171.9 ± 1,658.8 mL, with no observed differences between subgroups.

ROC analysis revealed that AUCs of anaesthesia time, surgery time, bleeding volume, and intake/output were 0.745, 0.726, 0.737, or 0.585, respectively, which are poor predictive values (AUC 0.5–0.75) for AKI (Supplementary Fig. 1).

### Clinical outcomes

ICU length of stay (LOS) was longer in patients with AKI (8.0 ± 8.7 days vs 3.3 ± 2.3 days, *p* = 0.003), although hospital LOS did not differ significantly. Two patients with AKI died, but none required renal replacement therapy. None of the patients in the AKI group required renal replacement therapy.

### Biomarker analysis

In the 25 patients who did not develop AKI, no significant increase was noted in the concentrations of urinary biomarkers, including [TIMP-2]•[IGFBP7], TIMP-2, IGFBP7, NGAL, and L-FABP, at any time point after ICU admission compared with the preoperative measurement. Conversely, those who subsequently developed AKI showed a striking increase in the concentration of urinary biomarkers at certain time points compared with preoperative measurements. The pattern of urinary biomarker excretion in patients with AKI was characterised by a peak very early after the precipitating event, followed by a decrease.

**Fig. 2:**
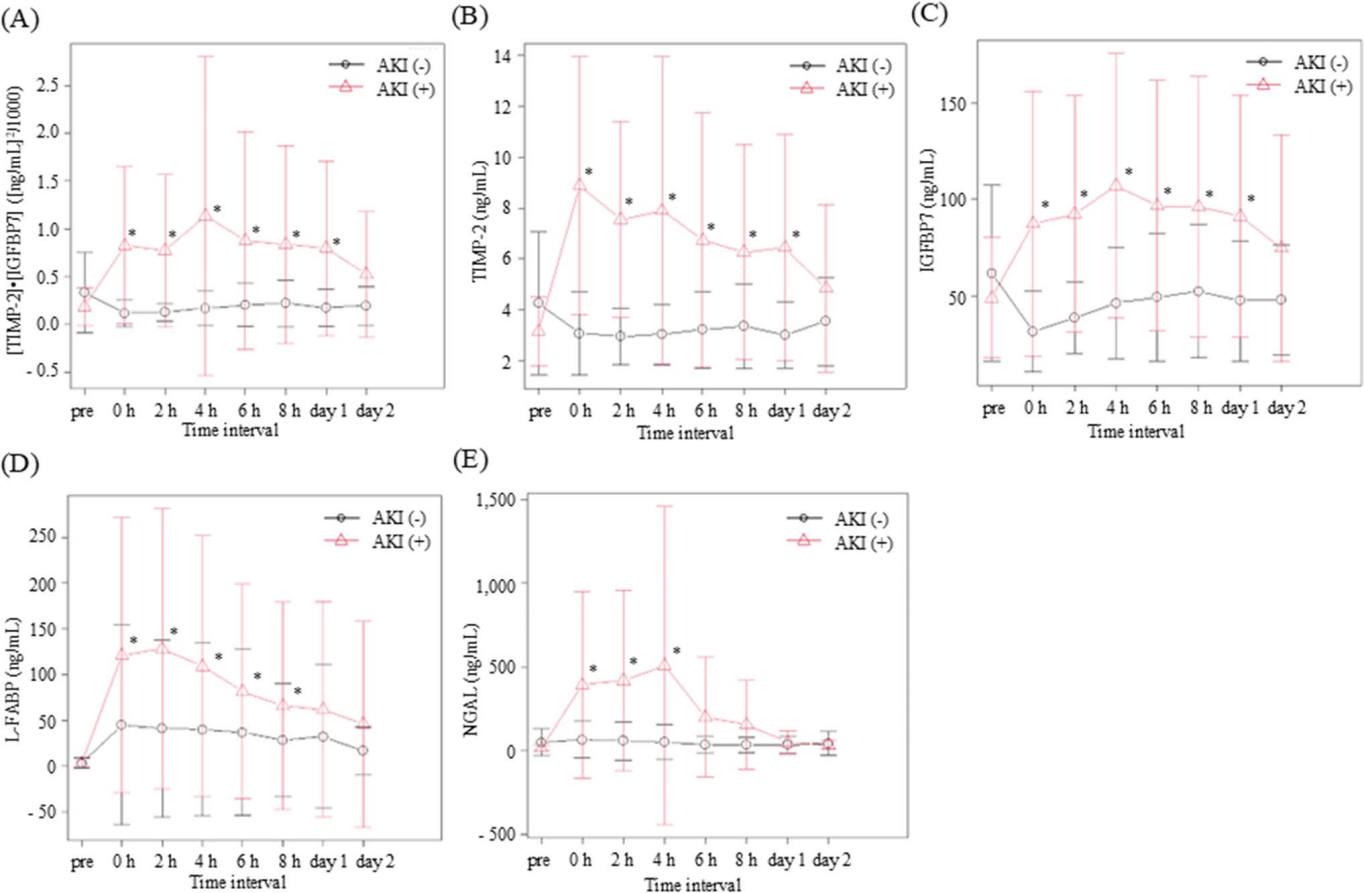
Analysis of urine [TIMP-2]•[IGFBP7], TIMP-2, IGFBP7, L-FABP, and NGAL. **A** Mean urine [TIMP-2]•[IGFBP7] concentrations in urine samples collected preoperatively and 0 h, 2 h, 4 h, 6 h, 8 h, 1 d, and 2 days after ICU admission. **B** Mean urine TIMP-2 concentrations of urine [TIMP-2]•[IGFBP7] in urine samples collected preoperatively and at 0 h, 2 h, 4 h, 6 h, 8 h, 1 d, and 2 days after ICU admission. **C** Mean urine IGFBP7 concentrations and mean urine [TIMP-2]•[IGFBP7] concentrations in urine samples collected preoperatively and at 0 h, 2 h, 4 h, 6 h, 8 h, 1 d, and 2 days after ICU admission. **D** Mean urine L-FABP concentrations and mean urine [TIMP-2]•[IGFBP7] concentrations in urine samples collected preoperatively and at 0 h, 2 h, 4 h, 6 h, 8 h, 1 d, and 2 days after ICU admission. **E** Mean urine NGAL concentrations of urine [TIMP-2]•[IGFBP7] in urine samples collected preoperatively and at 0 h, 2 h, 4 h, 6 h, 8 h, 1 d, and 2 days after ICU admission. Error bars are SD. Asterisks (*) denote significant differences (*p* < 0.05) between groups (AKI, non-AKI) at the respective time point. *AKI* acute kidney injury; IGFBP7 insulin-like growth factor-binding protein 7; *L-FABP* L-type fatty acid-binding protein; *NGAL* neutrophil gelatinase-associated lipocalin; *TIMP-2* tissue inhibitor of metalloproteinase 2

In patients who developed AKI compared to those who did not, urinary [TIMP-2]•[IGFBP7], TIMP-2, and IGFBP7 levels significantly increased from 0 h to day 1 after ICU admission (Fig. 2A–2C, Supplementary Table 1). Urinary L-FABP significantly increased from 0 to 8 h after ICU admission (Fig. 2D, Supplementary Table 1), and urinary NGAL significantly increased from 0 to 4 h after ICU admission (Fig. 2E, Supplementary Table 1). As shown in Supplementary Table 1, [TIMP-2]•[IGFBP7], TIMP-2, IGFBP7, NGAL, and L-FABP levels were significantly higher than the preoperative baseline values at 0–8 h, 0–8 h, 2–8 h and day 1, 0–6 h and day 1, day 2, or 0 h to day 2, respectively.

Table 2 summarises the sensitivity and specificity analyses using ROC curves for predicting any stage of AKI using [TIMP-2]•[IGFBP7], TIMP-2, IGFBP7, NGAL, and L-FABP levels.

**Table 2:** Prediction of postoperative AKI using biomarkers by ROC curve

Variable	AUC (95%CI)	Cut-off	Sensitivity	Specificity
[TIMP-2]•[IGFBP7]		[ng/mL]^2^/1000		
Preoperative	0.440 (0.242–0.638)	0.181	0.462	0.560
0 h after ICU admission	0.908 (0.809–1.000)	0.146	0.923	0.840
2 h after ICU admission	0.932 (0.835–1.000)	0.292	0.846	0.960
4 h after ICU admission	0.908 (0.809–1.000)	0.272	0.846	0.880
6 h after ICU admission	0.801 (0.646–0.956)	0.156	0.923	0.708
8 h after ICU admission	0.765 (0.592–0.938)	0.152	0.846	0.680
Day 1 after ICU admission	0.758 (0.573–0.944)	0.173	0.769	0.720
Day 2 after ICU admission	0.597 (0.366–0.826)	0.357	0.500	0.857
TIMP-2		(ng/mL)		
Preoperative	0.432 (0.240–0.624)	2.513	0.769	0.400
0 h after ICU admission	0.902 (0.798–1.000)	4.108	0.846	0.800
2 h after ICU admission	0.914 (0.795–1.000)	4.218	0.923	0.880
4 h after ICU admission	0.917 (0.816–1.000)	5.208	0.846	0.960
6 h after ICU admission	0.821 (0.673–0.968)	4.241	0.692	0.792
8 h after ICU admission	0.763 (0.588–0.938)	2.756	0.846	0.640
Day 1 after ICU admission	0.782 (0.610–0.953)	3.838	0.692	0.760
Day 2 after ICU admission	0.569 (0.332–0.807)	5.758	0.500	0.905
IGFBP7		(ng/mL)		
Preoperative	0.437 (0.237–0.637)	51.276	0.538	0.520
0 h after ICU admission	0.838 (0.698–0.978)	30.316	0.923	0.720
2 h after ICU admission	0.851 (0.696–1.000)	59.972	0.769	0.880
4 h after ICU admission	0.852 (0.705–1.000)	76.965	0.769	0.880
6 h after ICU admission	0.792 (0.628–0.955)	55.146	0.846	0.708
8 h after ICU admission	0.748 (0.571–0.924)	66.166	0.692	0.760
Day 1 after ICU admission	0.749 (0.565–0.933)	65.594	0.692	0.800
Day 2 after ICU admission	0.623 (0.400–0.847)	48.370	0.583	0.667
NGAL		(ng/mL)		
Preoperative	0.345 (0.156–0.533)	15.1	0.385	0.560
0 h after ICU admission	0.748 (0.568–0.927)	11.9	0.846	0.680
2 h after ICU admission	0.760 (0.591–0.929)	14.1	0.769	0.640
4 h after ICU admission	0.711 (0.533–0.889)	13.7	0.692	0.640
6 h after ICU admission	0.628 (0.424–0.832)	21.7	0.538	0.708
8 h after ICU admission	0.629 (0.431–0.828)	18.1	0.615	0.640
Day 1 after ICU admission	0.629 (0.434–0.825)	17.7	0.615	0.640
Day 2 after ICU admission	0.552 (0.347–0.756)	16.0	0.833	0.429
L–FABP		(ng/mL)		
Preoperative	0.480 (0.277–0.683)	1.420	0.692	0.400
0 h after ICU admission	0.774 (0.622–0.926)	2.550	0.846	0.640
2 h after ICU admission	0.782 (0.631–0.932)	26.190	0.615	0.880
4 h after ICU admission	0.751 (0.584–0.918)	21.120	0.615	0.800
6 h after ICU admission	0.744 (0.581–0.906)	7.970	0.769	0.625
8 h after ICU admission	0.745 (0.580–0.909)	9.910	0.692	0.720
Day 1 after ICU admission	0.671 (0.482–0.860)	11.480	0.615	0.760
Day 2 after ICU admission	0.575 (0.359–0.792)	10.130	0.500	0.714

*AKI* acute kidney injury, *AUC* area under the curve, *CI* confidence interval, *ICU* intensive care unit, *ROC* receiver operating characteristic, *TIMP-2* tissue inhibitor of metalloproteinase 2, *IGFBP7* insulin-like growth factor-binding protein 7, *NGAL* neutrophil gelatinase-associated lipocalin, *L-FABP* L-type fatty acid-binding protein

**Fig. 3:**
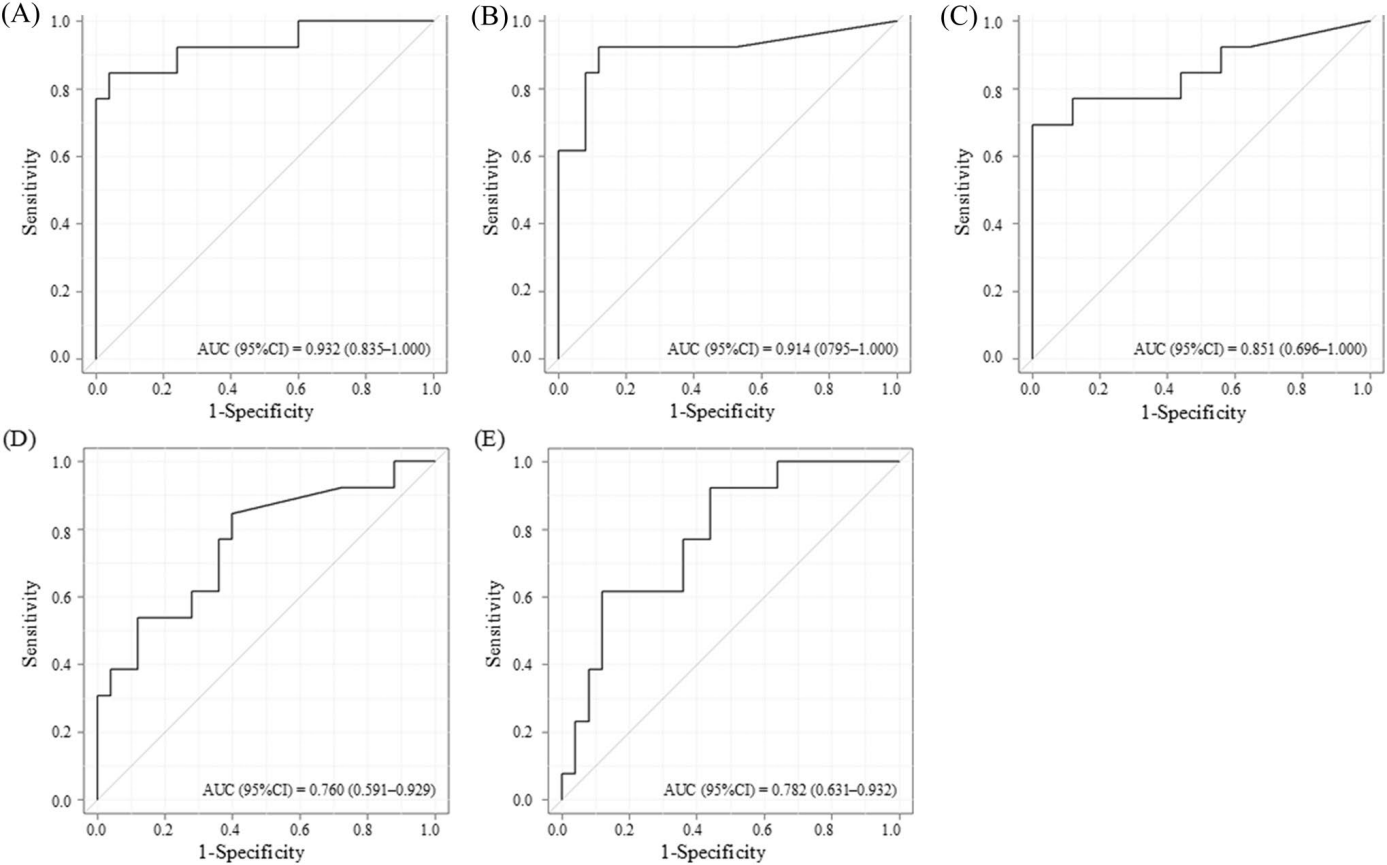
ROC curves for predicting any stage of AKI using [TIMP-2]•[IGFBP7] (**A**), TIMP-2 (**B**), IGFBP7 (**C**), NGAL (**D**), or L-FABP (**E**) results from urine samples collected 2 h after ICU admission. *AKI* acute kidney injury; *AUC* area under the curve; *CI* confidence interval; *ROC*, receiver operating characteristic; *TIMP-2* tissue inhibitor of metalloproteinase 2; *IGFBP7*, insulin-like growth factor-binding protein 7; *NGAL* neutrophil gelatinase-associated lipocalin; L-*FABP* L-type fatty acid-binding protein

ROC analysis revealed that [TIMP-2]•[IGFBP7] and TIMP-2 had excellent predictive values (AUC > 0.9) for AKI from 0 to 4 h after ICU admission and good predictive values (AUC 0.75–0.9) for AKI from 6 h to day 1 after ICU admission (Table 2, Supplementary Fig. 2, 3). IGFBP7 had a good predictive value (AUC 0.75–0.9) for AKI from 0 to 6 h after ICU admission (Table 2, Supplementary Fig. 4), while L-FABP had a good predictive value (AUC 0.75–0.9) for AKI from 0 to 4 h after ICU admission, and NGAL had a good predictive value (AUC 0.75–0.9) for AKI from 0 to 2 h after ICU admission (Table 2, Supplementary Fig. 5, 6). Biomarker levels preoperatively and on day 2 after ICU admission had poor or no diagnostic value (AUC < 0.75). The highest predictive accuracy for all biomarkers was observed 2 h after ICU admission. AUC values for 2 h after ICU admission were the highest in the following order: [TIMP-2]•[IGFBP7] (0.932), TIMP-2 (0.914), IGFBP7 (0.851), L-FABP (0.782), and NGAL (0.760) (Fig. 3). The corresponding cut-off values were 0.292 [ng/mL]^2^/1,000, 4.218 ng/mL, 59.972 ng/mL, 26.190 ng/mL, and 14.1 ng/mL, respectively.

## Discussion

Our study evaluated the diagnostic value of the urinary biomarkers [TIMP-2]•[IGFBP7], TIMP-2, IGFBP7, NGAL, and L-FABP for predicting CSA–AKI. The highest predictive accuracy occurred at 0–4 h after ICU admission, with [TIMP-2]•[IGFBP7] showing the best performance at 2 h. Approximately one-third of patients (34.2%) undergoing cardiovascular surgery developed AKI, with stage 1, 2, and 3 rates of 18.4%, 10.5%, and 5.2%, respectively, aligning with previous studies [19, 20].

Recent studies have explored various biomarkers for early detection of AKI. Tubular injury biomarkers, such as NGAL and L-FABP, alongside cell cycle arrest biomarkers, such as TIMP2 and IGFBP7, have demonstrated potential in both animal models and initial human research [21]. NGAL is the most studied AKI biomarker, reabsorbed by the proximal tubules and released by damaged distal tubules during acute tubular injury, and detectable within h, even without functional AKI [19, 22]. Previous studies have reported that, in humans, elevated NGAL levels can increase within 2–3 h after tubular injury and peak around 6–12 h, depending on the severity of the injury [19, 23, 24]. L-FABP is also expressed in the kidney and is a frequently studied biomarker of AKI. L-FABP has a high affinity and binding capacity for long-chain fatty acid oxidation products, suggesting that it could be a potent endogenous antioxidant [19, 25]. A previous report showed that urinary L-FABP concentration is utilised as an early and sensitive predictor of paediatric CSA–AKI at 4 h postoperatively [26]. Urine NGAL and L-FABP levels exhibited composite AUCs of 0.69–0.72 for predicting CSA–AKI in adults [21]. Levels of urinary IGFBP7 and TIMP-2, two biomarkers involved in G1 cell cycle arrest [11], accurately predicted CSA–AKI [15]. Previous studies have suggested that the urinary product of TIMP-2 and IGFBP7 may have a renoprotective effect, consistent with its role in an epithelial defence mechanism via the induction of G1 cell cycle arrest [27]. These urinary cell-cycle arrest markers reflect renal tubular stress in the early stages of AKI [11]. The combined biomarker [TIMP-2]•[IGFBP7] has demonstrated strong predictive ability for AKI in large, diverse cohorts, including the Sapphire, Opal, and Topaz studies [11, 13, 14]. Additionally, several studies have evaluated its performance in predicting AKI in patients undergoing cardiac surgery [13, 14, 20, 28]. In these populations, postoperative urinary [TIMP-2]•[IGFBP7] has shown predictive values with an AUC as high as 0.9. Furthermore, urinary [TIMP-2]•[IGFBP7] outperforms traditional biomarkers such as SCr, NGAL, and L-FABP in AKI prediction. In a subgroup analysis of the Sapphire and Topaz studies, urinary [TIMP-2]•[IGFBP7] predicted AKI with an AUC of 0.84 in patients who underwent cardiac surgery [29]. Our findings align with previous reports and contribute to a better understanding of the optimal timing for measuring these novel biomarkers. We suggest that biomarkers like [TIMP-2]•[IGFBP7] can detect early renal injury stress, potentially bridging the diagnostic and treatment delay, even when using KDIGO criteria.

The primary endpoint of many studies on CSA–AKI is to detect AKI stages 2–3 [30]. Certainly, in the short term, severe CSA–AKI is independently associated with a 3–eightfold higher perioperative mortality, prolonged hospital and ICU LOS, and increased cost of care [31]. However, in the long term, CSA–AKI is independently associated with an increased risk of end-stage renal disease and death [5, 32]. Furthermore, previous studies have reported that more than 90% of patients with CSA–AKI have AKI stage 1, and worse outcomes have been reported in these patients compared to those without AKI [33]. AKI is a syndrome that was proposed based on the recognition that even a slight increase in SCr, defined as AKI stage 1, affects long-term renal and life prognosis [8]. Our study demonstrated that biomarkers such as [TIMP-2]•[IGFBP7], TIMP-2, IGFBP7, NGAL, and L-FABP effectively predicted short-term events influenced by AKI stages 2–3 and the full AKI spectrum, including stage 1, which constitutes the majority of CSA–AKI cases. We recommend that future research and therapeutic targeting consider this often-overlooked aspect in CSA–AKI studies.

SCr, blood urea nitrogen, and urine volume are traditional biomarkers of renal function, but they have limited ability to predict the risk of renal injury. By definition, declining renal function indicates renal injury, leading to the accumulation of end products of nitrogen metabolism (e.g., urea and creatinine) in the circulation. When measured serially, a small increase in creatinine level defines AKI, at which point renal injury occurs. In comparison, [IGFBP7]•[TIMP-2] is not an indicator of declining renal function but rather an indicator of early signs of renal stress, assessing the risk of renal injury.

Recently, the concept of "subclinical AKI" has been proposed for the early diagnosis of structural changes in renal tissues using biomarkers before a decline in renal function is detected by elevated SCr levels [19]. The human kidney has significant functional reserve, meaning that kidney dysfunction may not become apparent until more than 50% of the kidney mass is compromised, resulting in a greater than 50% decrease in the glomerular filtration rate (GFR). Consequently, relying solely on changes in SCr levels to detect kidney injury may lead to delayed diagnosis, causing the clinical "window of opportunity" for timely treatment to be missed, particularly in cases of AKI [19]. AKI diagnosed only by elevated markers of tubular or glomerular injury is called subclinical AKI, and it is imperative to improve the recognition of subclinical AKI when a clinical syndrome is characterised by normal clinical SCr levels and normal or mildly decreased GFR [19]. The diagnostic process for AKI should be revised to incorporate both markers of renal function, such as changes in SCr and UO, and markers of tubular injury. This approach would improve the characterisation of AKI phenotypes, enhance diagnostic accuracy, and enable the detection of kidney damage before SCr levels rise, facilitating the identification and treatment of subclinical AKI [19]. Based on our results, tubular injury markers and cell cycle arrest markers, such as [TIMP-2]•[IGFBP7], TIMP-2, IGFBP7, NGAL, and L-FABP, are expected to be powerful tools for diagnosing subclinical AKI.

Many pharmacological and non-pharmacological interventions have been tested to reduce the incidence of CSA–AKI. However, randomised controlled trials (RCTs) have presented inconsistent results [34]. Several meta-analyses have been conducted; however, none have demonstrated the superiority of a particular intervention in reducing the incidence of CSA–AKI [35–37]. The failure of these studies may be attributed to the timing of biomarker measurement. In several cases, measurements were taken much later than the proposed optimal window of 0–4 h, with therapeutic interventions delayed even further, resulting in postponed diagnosis and treatment. However, there are concerns regarding the effectiveness of these interventions. Prior research has not rigorously examined the optimal timing for [TIMP-2]•[IGFBP7] measurement in diagnosing AKI as comprehensively as in our study. We suggest that future [TIMP-2]•[IGFBP7] measurements for accurately diagnosing AKI, including CSA–AKI, should be conducted within 4 h of the traumatic event, ideally at the 2-h mark.

Only a few studies have focused on the Asian population, with one investigation examining urinary [TIMP-2]•[IGFBP7] for predicting CSA–AKI [38] and another investigating urinary TIMP-2 for predicting AKI, primarily in septic patients in ICU settings [39]. Wang et al. reported that 20 (35%) of 57 patients who underwent cardiac surgery developed CSA–AKI. The AUC from ROC analysis of [TIMP-2]•[IGFBP7] within 4 h of ICU admission for predicting the onset of CSA–AKI was 0.80 [38]. The incidence of AKI in their study was consistent with ours, suggesting that [TIMP-2]•[IGFBP7] is effective for predicting CSA–AKI. However, unlike our study, only [TIMP-2]•[IGFBP7] was analysed, and no comparison was made with other AKI biomarkers, such as NGAL and L-FABP. Additionally, the time course of [TIMP-2]•[IGFBP7] in their study showed that the average value was twice as high as preoperative levels 2 h after ICU admission, which is consistent with our finding that all AKI biomarkers were significantly elevated 2 h post-admission compared to preoperative levels. Yamashita et al. investigated the effectiveness of urinary TIMP-2 and plasma NGAL in predicting AKI in a cohort of 98 Japanese ICU patients, 42 (42.9%) of whom developed AKI [39]. The AUCs for predicting AKI using urinary TIMP-2 and plasma NGAL were 0.75 and 0.84, respectively. When focusing on septic AKI, the AUCs were 0.78 and 0.94, respectively. Similar to our study, they found urinary TIMP-2 effective in predicting AKI in Japanese patients; however, they did not examine or compare urinary [TIMP-2]•[IGFBP7], urinary IGFBP7, urinary NGAL, or urinary L-FABP. Furthermore, serum and urine samples in their study were collected at ICU admission. Their patient cohort was heterogeneous, with AKI primarily caused by sepsis, although other conditions were also involved, differing from the CSA–AKI patients in our study. This finding suggests that the timing of true renal injury events is heterogeneous, unlike studies similar to ours, where the timing of cardiovascular surgery was precisely defined. We emphasise the significance of our study, as it compares the effectiveness of urinary [TIMP-2]•[IGFBP7], TIMP-2, IGFBP7, NGAL, and L-FABP in predicting CSA–AKI in Asian populations and investigates the optimal timing for measuring these biomarkers.

Our study had limitations, including being a single-centre study with a small sample size, which may limit the generalizability of our findings. Additionally, we did not fully consider the various techniques and approaches used in cardiovascular surgery. Further research is needed on CSA–AKI in patients undergoing diverse cardiovascular surgeries. However, the identification of the optimal timing for biomarker measurement in this study is expected to advance CSA–AKI research.

## Conclusions

Our study demonstrated that urine biomarkers, including [TIMP-2]•[IGFBP7], TIMP-2, IGFBP7, NGAL, and L-FABP, provided good predictive performance for any stage of CSA–AKI, with [TIMP-2]•[IGFBP7] showing the best performance. The optimal timing for measuring urinary [TIMP-2]•[IGFBP7] to diagnose CSA–AKI is 0–4 h after ICU admission, with 2 h post-admission being optimal. Our findings suggest that [TIMP-2]•[IGFBP7] is particularly useful for diagnosing CSA–AKI in Asian populations, including Japanese patients, and that its optimal diagnostic window is earlier than previously reported. Future studies should incorporate larger, multicenter cohorts to validate our findings across diverse populations and surgical methods in patients undergoing cardiovascular surgery. This will help to better understand the potential of these biomarkers for the early detection and management of CSA–AKI.

## Supplementary Information

Below is the link to the electronic supplementary material.

Supplementary file1 (TIF 4141 KB)

Supplementary file2 (TIF 5294 KB)

Supplementary file3 (TIF 5363 KB)

Supplementary file4 (TIF 5338 KB)

Supplementary file5 (TIF 5358 KB)

Supplementary file6 (TIF 5285 KB)

Supplementary file7 (DOCX 27 KB)

## Data Availability

The ethics committee of Kochi Medical School Hospital restricts the sharing of clinical data, as these are confidential and are subject to general data protection regulations. The datasets used and/or analysed during the current study are available from the corresponding authors upon reasonable request.
