# Supplementary figures and images for: Urinary [TIMP-2]•[IGFBP7], TIMP-2, IGFBP7, NGAL, and L-FABP for the prediction of acute kidney injury following cardiovascular surgery in Japanese patients
Source: Clin Exp Nephrol. 2025 Apr 7;29(9):1172–82. doi: 10.1007/s10157-025-02671-2 (PMC12441090; doi:10.1007/s10157-025-02671-2)

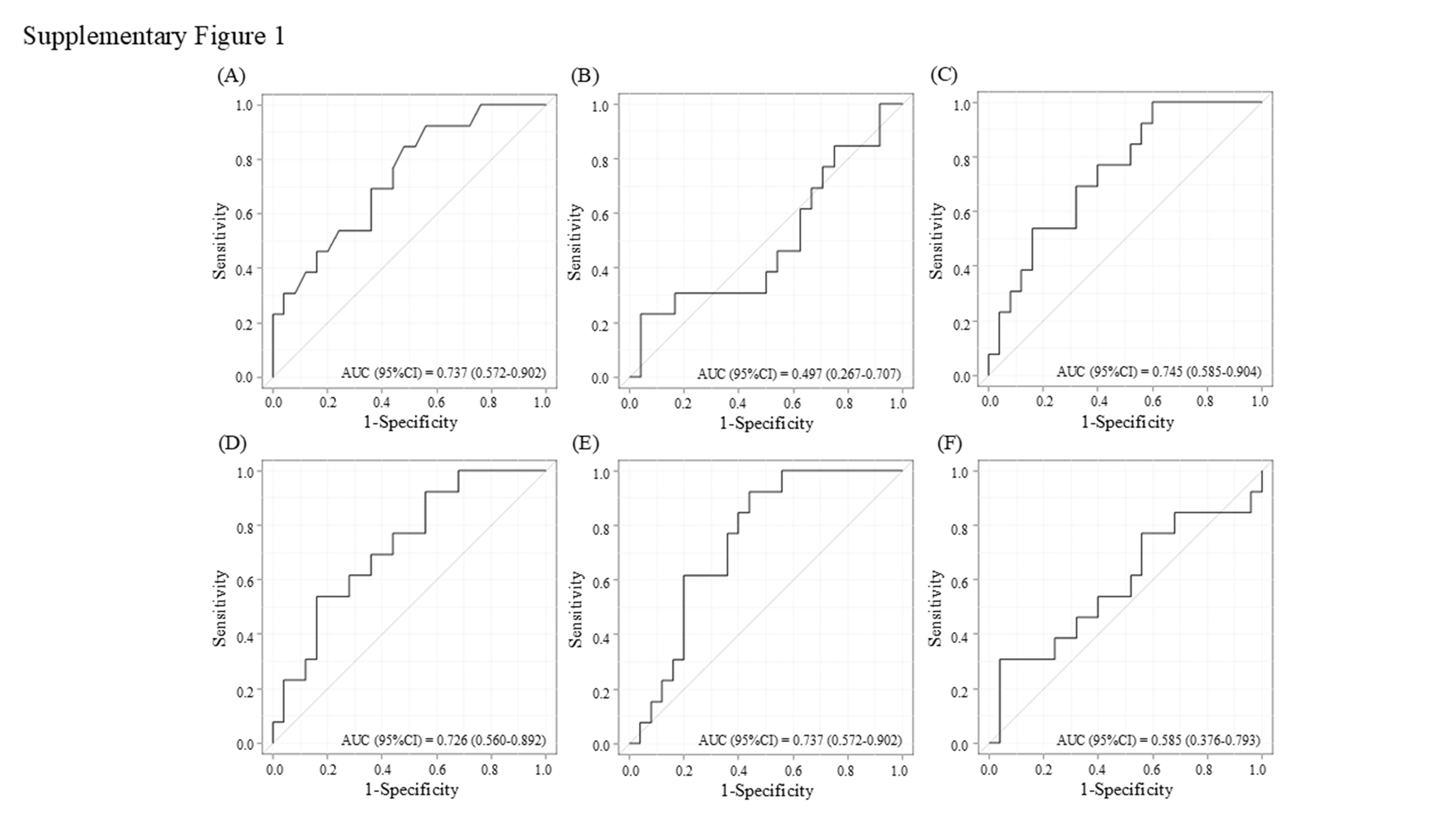

Supplement: Supplementary file 1 — Supplementary file1 (TIF 4141 KB) [file 10157_2025_2671_MOESM1_ESM.tif]

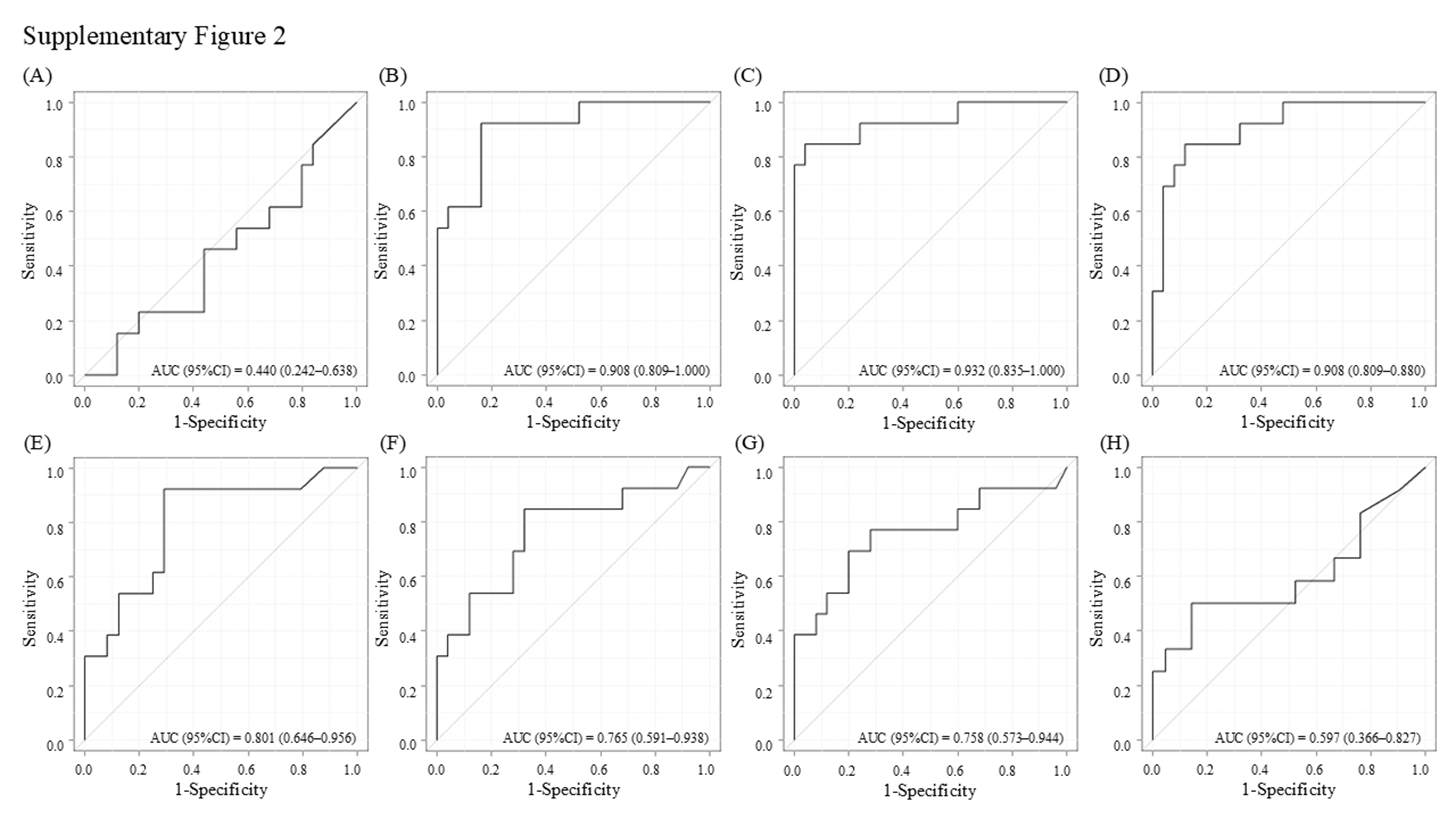

Supplement: Supplementary file 2 — Supplementary file2 (TIF 5294 KB) [file 10157_2025_2671_MOESM2_ESM.tif]

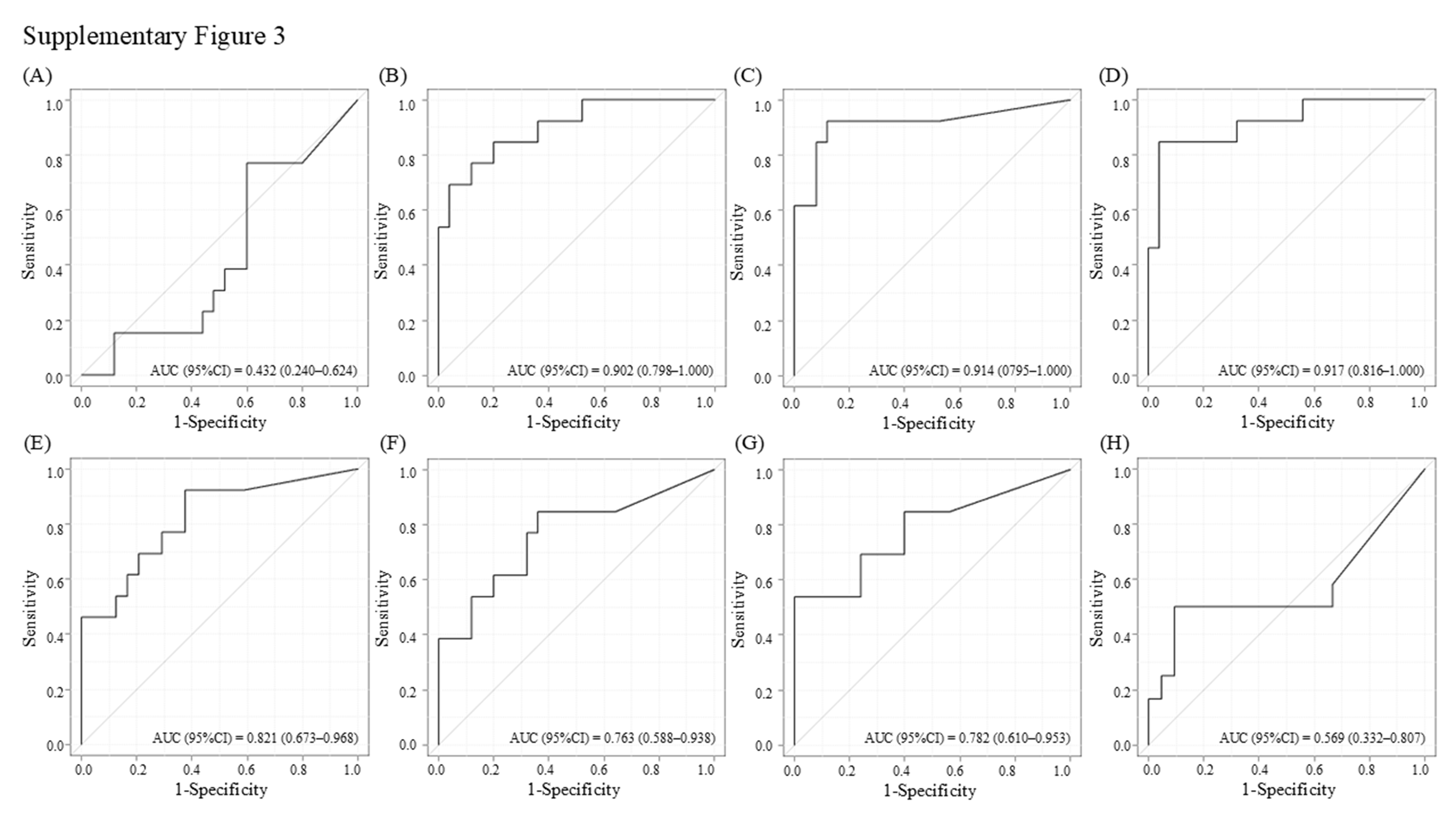

Supplement: Supplementary file 3 — Supplementary file3 (TIF 5363 KB) [file 10157_2025_2671_MOESM3_ESM.tif]

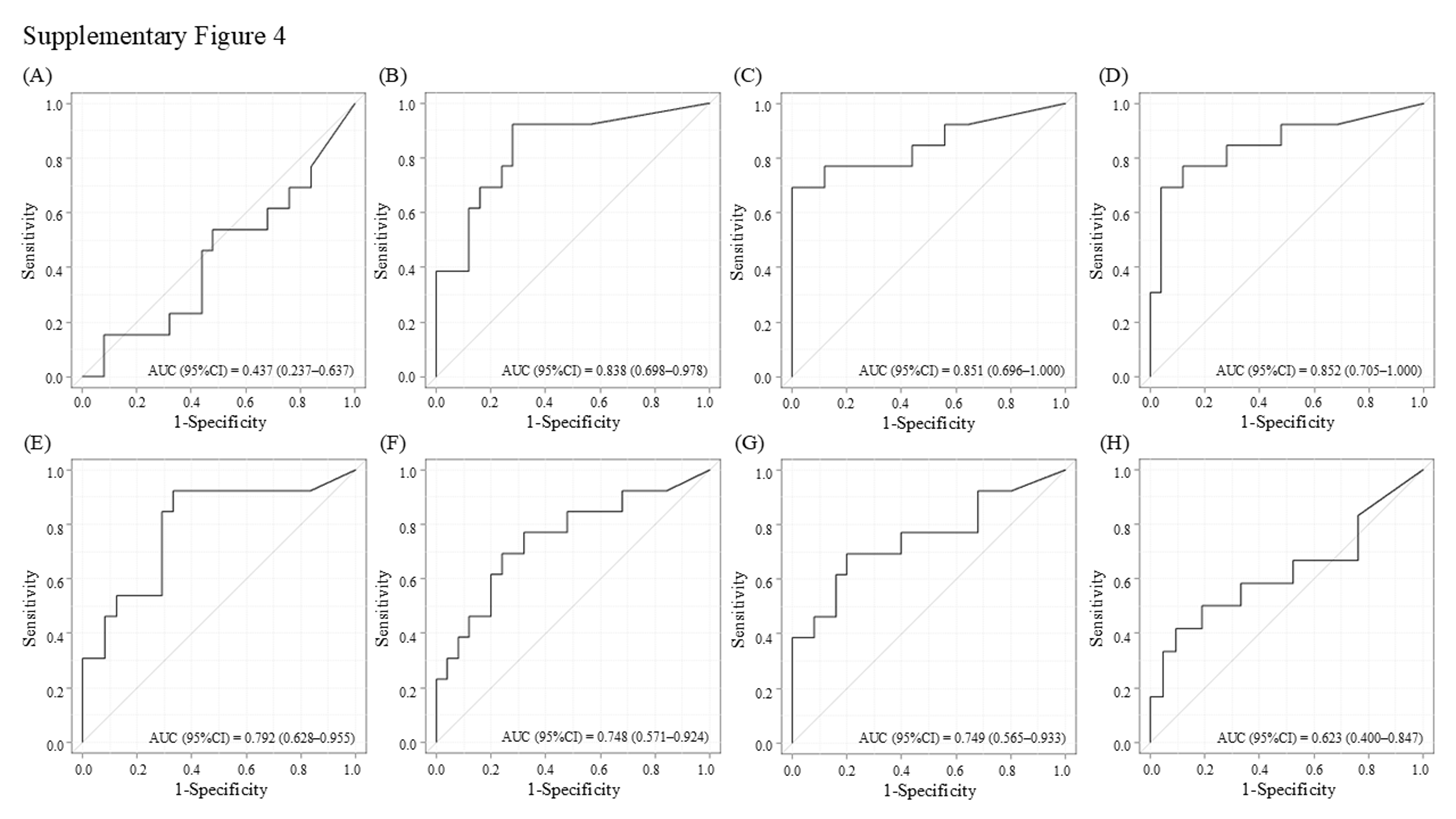

Supplement: Supplementary file 4 — Supplementary file4 (TIF 5338 KB) [file 10157_2025_2671_MOESM4_ESM.tif]

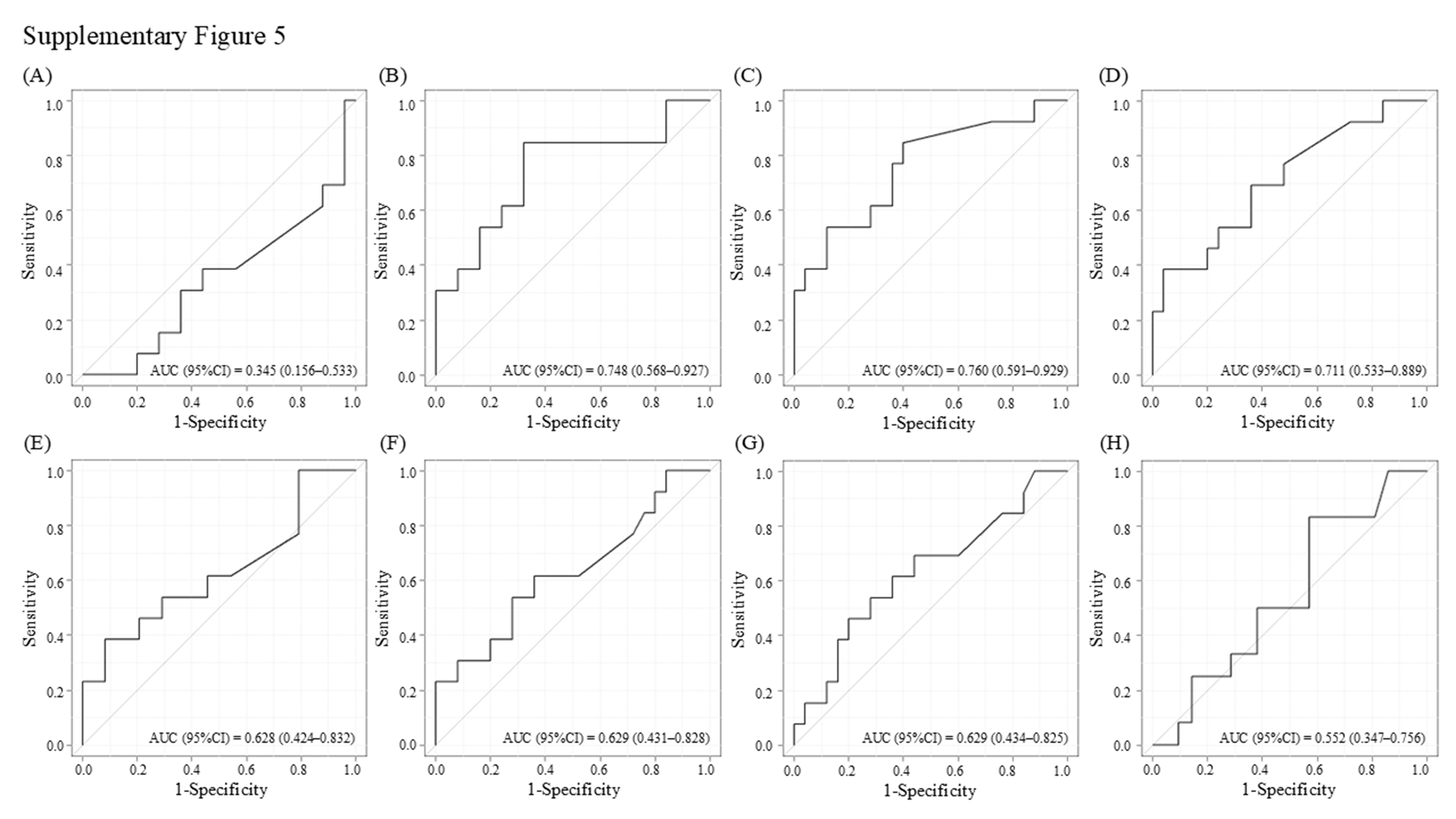

Supplement: Supplementary file 5 — Supplementary file5 (TIF 5358 KB) [file 10157_2025_2671_MOESM5_ESM.tif]

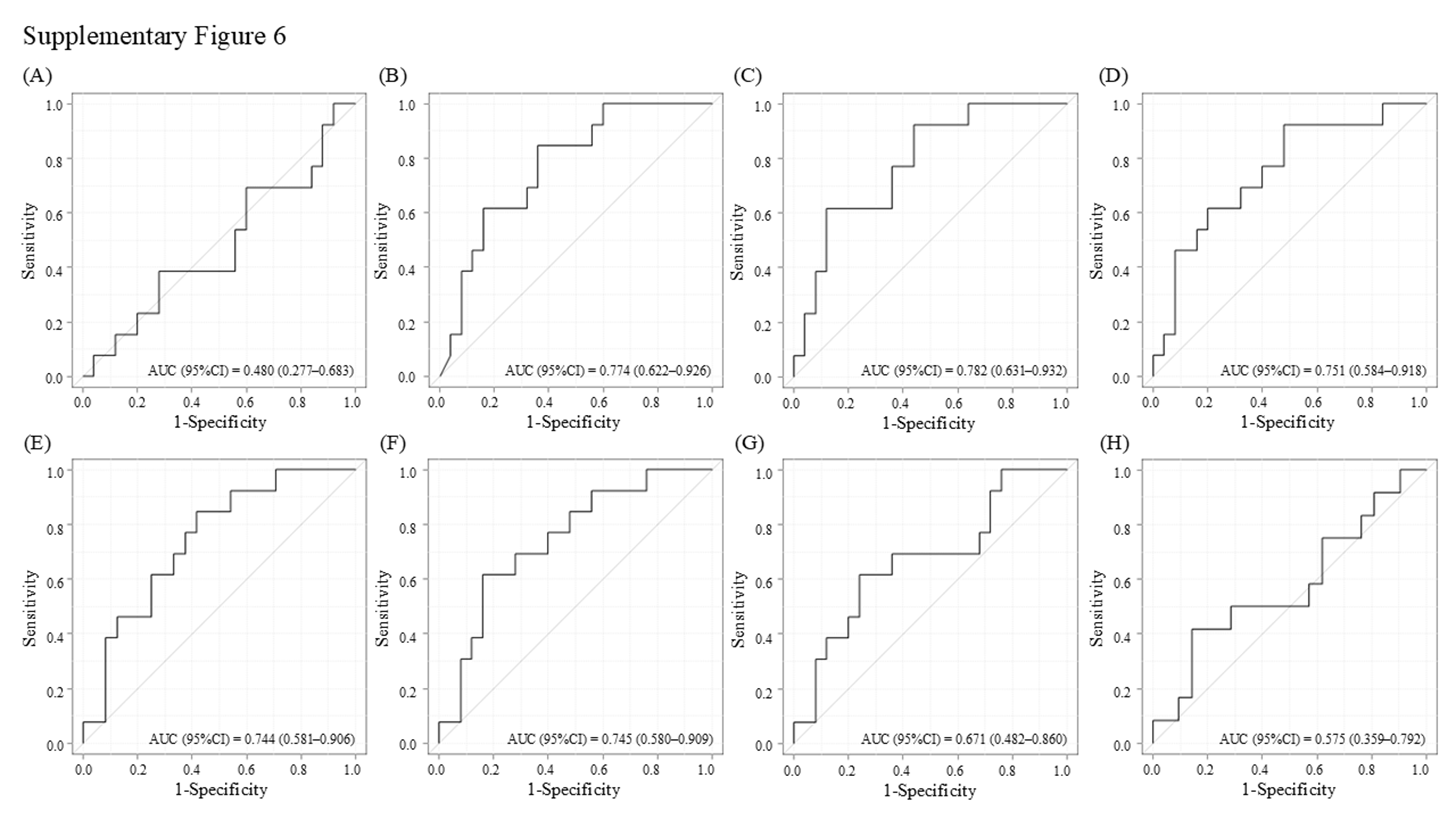

Supplement: Supplementary file 6 — Supplementary file6 (TIF 5285 KB) [file 10157_2025_2671_MOESM6_ESM.tif]
