# Supplementary material for: Urinary [TIMP-2]•[IGFBP7], TIMP-2, IGFBP7, NGAL, and L-FABP for the prediction of acute kidney injury following cardiovascular surgery in Japanese patients
Source: Clin Exp Nephrol. 2025 Apr 7;29(9):1172–82. doi: 10.1007/s10157-025-02671-2 (PMC12441090; doi:10.1007/s10157-025-02671-2)
Supplement: Supplementary file 7 — Supplementary file7 (DOCX 27 KB) [file 10157_2025_2671_MOESM7_ESM.docx]

***Clinical and Experimental Nephrology***

**Urinary [TIMP-2]•[IGFBP7], TIMP-2, IGFBP7, NGAL, and L-FABP for the prediction of acute kidney injury following cardiovascular surgery in Japanese patients**

Hideki Iwata^1^, MD, Taro Horino^2*^, MD, PhD, Yuki Osakabe^2^, MD, PhD, Satoshi Inotani^2^, MD, PhD, Keisuke Yoshida^3^, MD, Keita Mitani^4^, PhD, Yutaka Hatakeyama^4^, PhD, Yujiro Miura^3^, MD, PhD, Yoshio Terada^2^, MD, PhD, Takashi Kawano^1^, MD, PhD.

^1^Department of Anaesthesiology and Intensive Care Medicine, Kochi Medical School, Kochi University, Kohasu, Oko-cho, Nankoku, Kochi 783-8505, Japan

^2^Department of Endocrinology, Metabolism and Nephrology, Kochi Medical School, Kochi University, Kohasu, Oko-cho, Nankoku, Kochi 783-8505, Japan

^3^Department of Cardiovascular Surgery, Kochi Medical School, Kochi University, Kohasu, Oko-cho, Nankoku, Kochi 783-8505, Japan

^4^Centre of Medical Information Science, Kochi Medical School, Kochi University, Kohasu, Oko-cho, Nankoku, Kochi 783-8505, Japan

***Corresponding author:**

Taro Horino, MD, PhD

Department of Endocrinology, Metabolism and Nephrology, Kochi Medical School

Kochi University, Kohasu, Oko-cho, Nankoku, Kochi 783-8505, Japan

Phone: +81-88-880-2343;

Fax: +81-88-880-2344;

E-mail: [horinott@yahoo.co.jp](mailto:horinott@yahoo.co.jp)

Supplementary Table 1. Changes in biomarkers over time in subgroups with or without AKI.

| Variable, mean (SD) | AKI (n=13) | *p*-value | no AKI (n=25) | *p*-value | *p*-value |
| --- | --- | --- | --- | --- | --- |
|  |  | vs. Preoperative |  | vs. Preoperative | AKI vs. no AKI |
| [TIMP-2]•[IGFBP7] ([ng/mL]^2^/1000) | |  |  |  |  |
| Preoperative | 0.215 (0.217) |  | 0.321 (0.386) |  | 0.547 |
| 0 hour after ICU admission | 0.968 (0.942) | 0.005 | 0.122 (0.140) | 0.007 | <0.001 |
| 2 hours after ICU admission | 0.872 (0.840) | 0.007 | 0.107 (0.088) | 0.011 | <0.001 |
| 4 hours after ICU admission | 1.171 (1.599) | 0.004 | 0.143 (0.163) | 0.032 | <0.001 |
| 6 hours after ICU admission | 0.946 (1.116) | 0.011 | 0.173 (0.210) | 0.061 | 0.002 |
| 8 hours after ICU admission | 0.890 (1.007) | 0.029 | 0.195 (0.222) | 0.199 | 0.008 |
| Day 1 after ICU admission | 0.912 (0.965) | 0.064 | 0.156 (0.175) | 0.062 | 0.009 |
| Day 2 after ICU admission | 0.524 (0.654) | 0.445 | 0.182 (0.203) | 0.233 | 0.358 |
| TIMP-2 (ng/mL) |  |  |  |  |  |
| Preoperative | 3.523 (1.850) |  | 4.309 (2.715) |  | 0.496 |
| 0 hour after ICU admission | 9.501 (5.331) | 0.001 | 3.077 (1.557) | 0.034 | <0.001 |
| 2 hours after ICU admission | 8.009 (4.039) | 0.001 | 2.769 (1.050) | 0.009 | <0.001 |
| 4 hours after ICU admission | 8.152 (5.873) | 0.004 | 2.845 (1.117) | 0.025 | <0.001 |
| 6 hours after ICU admission | 7.151 (5.005) | 0.019 | 3.033 (1.448) | 0.050 | 0.001 |
| 8 hours after ICU admission | 6.625 (4.262) | 0.044 | 3.132 (1.565) | 0.079 | 0.007 |
| Day 1 after ICU admission | 7.070 (4.805) | 0.099 | 2.878 (1.212) | 0.021 | 0.004 |
| Day 2 after ICU admission | 4.855 (3.281) | 0.868 | 3.472 (1.725) | 0.322 | 0.502 |
| IGFBP7, (ng/mL) |  |  |  |  |  |
| Preoperative | 51.068 (30.646) |  | 60.368 (41.233) |  | 0.526 |
| 0 hour after ICU admission | 93.221 (68.783) | 0.117 | 33.480 (22.179) | 0.003 | <0.001 |
| 2 hours after ICU admission | 97.077 (61.018) | 0.033 | 35.755 (17.780) | 0.011 | <0.001 |
| 4 hours after ICU admission | 110.039 (66.386) | 0.009 | 43.268 (26.885) | 0.109 | <0.001 |
| 6 hours after ICU admission | 100.813 (63.611) | 0.033 | 47.081 (30.806) | 0.217 | 0.008 |
| 8 hours after ICU admission | 99.696 (65.612) | 0.029 | 50.671 (31.327) | 0.471 | 0.013 |
| Day 1 after ICU admission | 96.460 (62.570) | 0.048 | 45.436 (28.480) | 0.173 | 0.012 |
| Day 2 after ICU admission | 74.817 (58.555) | 0.428 | 46.666 (28.273) | 0.240 | 0.243 |
| NGAL, (ng/mL) |  |  |  |  |  |
| Preoperative | 15.0 (13.2) |  | 45.6 (75.0) |  | 0.115 |
| 0 hour after ICU admission | 374.1 (538.8) | 0.004 | 55.4 (100.1) | 0.400 | 0.012 |
| 2 hours after ICU admission | 390.8 (528.3) | 0.012 | 47.7 (103.6) | 0.304 | 0.008 |
| 4 hours after ICU admission | 473.7 (921.2) | 0.017 | 43.2 (92.4) | 0.415 | 0.034 |
| 6 hours after ICU admission | 188.6 (345.2) | 0.044 | 31.4 (45.9) | 0.648 | 0.200 |
| 8 hours after ICU admission | 147.2 (254.8) | 0.050 | 31.1 (42.4) | 0.537 | 0.194 |
| Day 1 after ICU admission | 52.1 (65.7) | 0.034 | 29.8 (46.7) | 0.646 | 0.195 |
| Day 2 after ICU admission | 32.0 (28.3) | 0.031 | 41.7 (69.5) | 0.492 | 0.626 |
| L–FABP, (ng/mL) |  |  |  |  |  |
| Preoperative | 2.873 (3.891) |  | 3.224 (5.170) |  | 0.841 |
| 0 hour after ICU admission | 117.682 (144.486) | 0.003 | 36.170 (98.392) | 0.793 | 0.006 |
| 2 hours after ICU admission | 120.788 (149.062) | 0.001 | 33.394 (87.707) | 0.472 | 0.004 |
| 4 hours after ICU admission | 102.349 (138.208) | 0.001 | 32.457 (85.290) | 0.171 | 0.012 |
| 6 hours after ICU admission | 76.537 (113.659) | <0.001 | 31.066 (83.449) | 0.045 | 0.015 |
| 8 hours after ICU admission | 61.883 (109.498) | <0.001 | 23.578 (55.784) | 0.006 | 0.014 |
| Day 1 after ICU admission | 58.167 (113.516) | <0.001 | 27.030 (70.599) | <0.001 | 0.087 |
| Day 2 after ICU admission | 45.923 (112.506) | 0.003 | 15.955 (25.690) | <0.001 | 0.477 |

AKI, acute kidney injury; SD, standard deviation; ICU, intensive care unit; ROC, receiver operating characteristic; TIMP-2, tissue inhibitor of metalloproteinase 2; IGFBP7, insulin-like growth factor-binding protein 7; NGAL, neutrophil gelatinase-associated lipocalin; L-FABP, L-type fatty acid-binding protein.
